# Endothelial glycocalyx dysfunction in the setting of mechanical thrombectomy: a conceptual link between cardiometabolic vulnerability and microvascular reperfusion failure

**DOI:** 10.3389/fneur.2026.1919445

**Published:** 2026-10-21

**Authors:** Xusheng Wu, Sufu Mi, Jing Ma, Yue Xing, Guangbin Li, Zongyu Cao, Xingjiang Xiong

**Affiliations:** 1 Clinical Medical College, Qinghai University, Xining, China; 2 Department of Cardiology, Guang'anmen Hospital, China Academy of Chinese Medical Sciences, Beijing, China

**Keywords:** acute ischemic stroke, blood–brain barrier, cardiometabolic vulnerability, endothelial glycocalyx, impaired microvascular reperfusion, mechanical thrombectomy, no-reflow, Syndecan-1

## Abstract

Mechanical thrombectomy (MT) has made macrovessel reopening achievable in acute ischemic stroke, but angiographic success does not ensure effective tissue-level reperfusion or microvascular oxygen delivery. This narrative review examines endothelial glycocalyx dysfunction as a plausible blood-facing interface that may connect pre-existing cardiometabolic vulnerability with post-thrombectomy microvascular dysfunction. Targeted English-language literature from PubMed/MEDLINE, Web of Science, Embase, and Google Scholar through 30 April 2026 was synthesized, with emphasis on MT, impaired microvascular reperfusion (IMR), no-reflow, blood–brain barrier (BBB) and neurovascular-unit (NVU) biology, glycocalyx-related biomarkers, cardiometabolic risk, perfusion imaging, and microcirculatory rescue. The evidence base is heterogeneous and is explicitly separated into direct human MT/LVO evidence, indirect human biomarker or imaging evidence, experimental cerebral evidence, non-cerebral/cross-disease vascular evidence, and hypothesis-generating mechanistic inference. Ischemic priming, abrupt reperfusion, oxidative and inflammatory stress, complement activation, metabolic stress, distal embolization, device-endothelial trauma, and host vulnerability may converge on the luminal endothelial surface. Glycocalyx dysfunction may amplify leukocyte adhesion and capillary plugging, platelet adhesion and microthrombosis, impaired mechanosensing and nitric-oxide signaling, BBB destabilization, and heterogeneous capillary flow; however, direct causal proof in human MT is lacking. Circulating syndecan-1, heparan sulfate, and hyaluronan are therefore best interpreted as time- and compartment-dependent systemic signals rather than direct readouts of the cerebral glycocalyx. Post-EVT microcirculatory rescue trials show that downstream perfusion may remain therapeutically actionable, but they do not demonstrate glycocalyx repair as the operative mechanism. A practical translational strategy is to combine serial, compartment-annotated biomarkers with prespecified early perfusion/barrier imaging and tissue endpoints, while explicitly modeling cardiometabolic vulnerability and competing microvascular mechanisms. The glycocalyx framework should therefore be considered a testable network hypothesis rather than an established primary cause of post-thrombectomy no-reflow or futile recanalization.

## Introduction: the post-thrombectomy reperfusion gap

Mechanical thrombectomy (MT) has transformed treatment of anterior-circulation large-vessel occlusion (LVO), and randomized evidence establishes the clinical value of endovascular therapy (EVT) across broad patient groups (1). Yet reopening the parent artery does not ensure restoration of effective parenchymal perfusion. Patients may achieve modified Thrombolysis in Cerebral Infarction/expanded Treatment in Cerebral Ischemia (mTICI/eTICI)-defined angiographic success and nevertheless experience persistent perfusion abnormalities, infarct growth, edema, hemorrhagic transformation (HT), or poor neurological recovery (2–5). This discrepancy is often summarized clinically as futile recanalization, but the biological pathway between technical success and patient-level outcome contains several distinct layers.

Parent-vessel patency, angiographic reperfusion, tissue-level perfusion, microvascular oxygen delivery, and clinical recovery are related but non-identical. The downstream microvascular bed may remain hypoperfused, become hyperperfused because of autoregulatory failure, exhibit capillary flow maldistribution or shunting, leak across the BBB, or become compressed by edema even when proximal flow has been restored (3–5). Accordingly, the present review focuses on the intermediate biological problem of incomplete tissue-level reperfusion rather than treating 90-day disability as a direct surrogate for a single microvascular mechanism.

The endothelial glycocalyx is a dynamic luminal surface layer composed of glycosaminoglycans, membrane-bound proteoglycans, glycoproteins, and associated plasma proteins (6–10). Its location places it at the interface between restored blood flow and the ischemia-primed endothelium. It can influence leukocyte and platelet contact, anticoagulant surface properties, mechanotransduction, permeability, and endothelial signaling. These features make glycocalyx dysfunction biologically plausible as one contributor to post-MT microvascular failure. The central claim of this review is deliberately narrow: glycocalyx dysfunction may amplify established microvascular injury pathways in susceptible patients; it should not be regarded as an established dominant cause of human MT-associated no-reflow or futile recanalization.

## Scope and narrative-review methodology

This article is a conceptual narrative review rather than a systematic review, meta-analysis, guideline, or consensus statement. The literature base was assembled through targeted searches of PubMed/MEDLINE, Web of Science, Embase, and Google Scholar for English-language publications available through 30 April 2026. Search concepts combined terms related to mechanical thrombectomy/endovascular therapy, impaired microvascular reperfusion/no-reflow, futile recanalization, endothelial glycocalyx, syndecan-1, heparan sulfate, hyaluronan, BBB/NVU biology, cardiometabolic vulnerability, diabetes, hyperglycemia, hypertension, dyslipidemia, renal dysfunction, inflammation, perfusion imaging, DSA parametric color coding (DSA-PCC), microcirculatory rescue, and intra-arterial thrombolysis. Reference lists of key reviews and primary studies were screened for additional mechanistically relevant reports. Searches were conducted as several targeted query families rather than as a single all-inclusive Boolean query; representative reproducible database-specific search strings reflecting these query families are provided in Supplementary Table S2. No PRISMA workflow, formal risk-of-bias assessment, or meta-analysis was performed because the objective was conceptual synthesis rather than systematic evidence grading. Generative-AI use was limited to language polishing and visual-layout assistance for Figures 1, 2. ChatGPT (OpenAI) was used for these limited purposes. Literature selection, evidence classification, interpretation, determination of mechanistic relationships, and scientific conclusions were performed by the authors, with details disclosed in the Acknowledgments.

**Figure 1:**
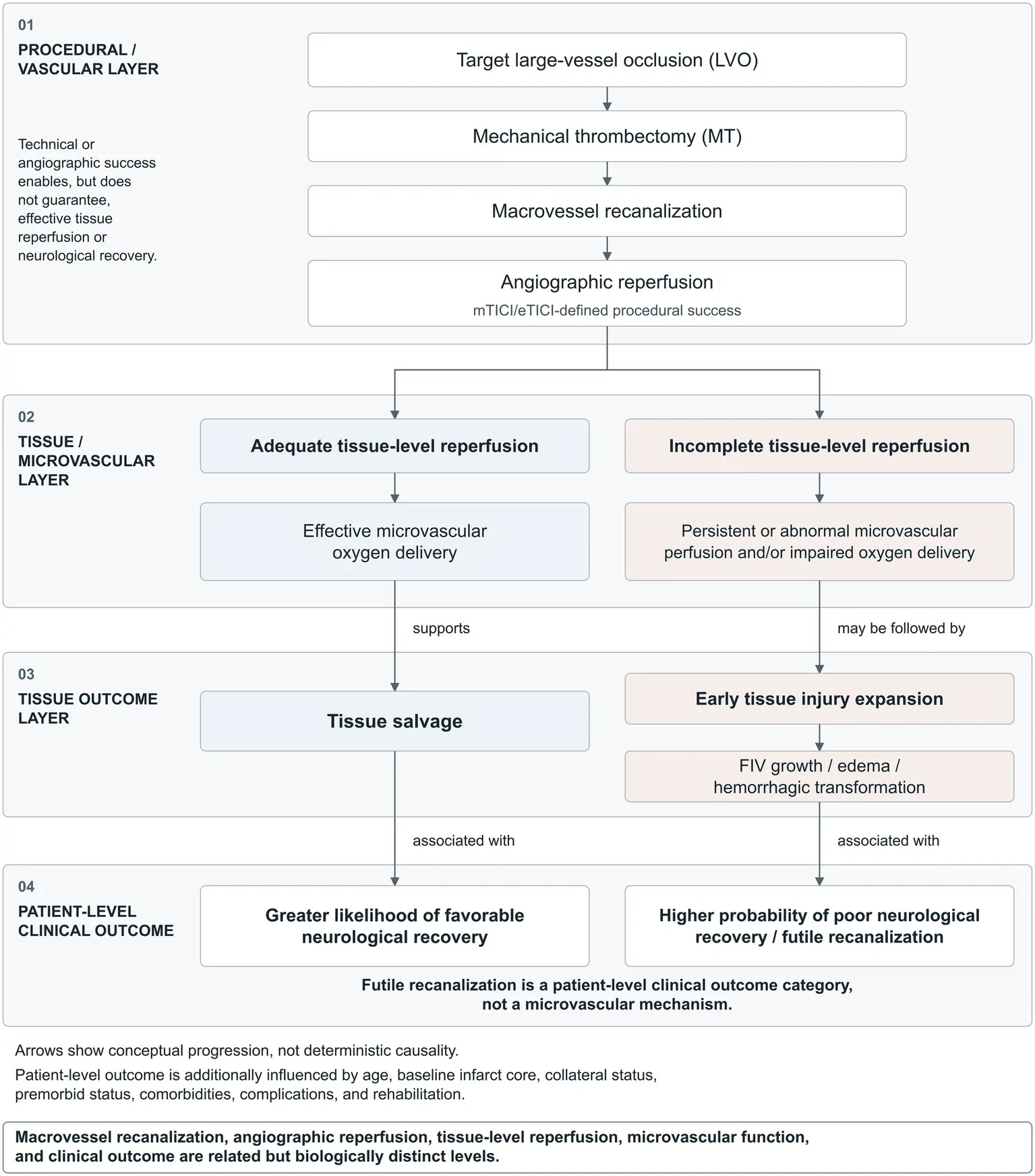
Conceptual hierarchy linking macrovessel recanalization, angiographic reperfusion, tissue-level reperfusion, and clinical outcome. The diagram separates the procedural/vascular, tissue/microvascular, tissue-outcome, and patient-level clinical layers. After mTICI/eTICI-defined angiographic success, adequate tissue-level reperfusion can support effective microvascular oxygen delivery and tissue salvage, whereas incomplete tissue-level reperfusion may coexist with persistent or abnormal microvascular perfusion and impaired oxygen delivery, followed by early tissue injury expansion, infarct growth or a larger final infarct volume (FIV), edema, and hemorrhagic transformation. Arrows indicate conceptual progression rather than deterministic causality. Patient-level outcome remains additionally influenced by age, baseline infarct core, collateral status, premorbid status, comorbidities, complications, and rehabilitation. Futile recanalization is a patient-level clinical outcome category, not a microvascular mechanism. LVO, large-vessel occlusion; MT, mechanical thrombectomy; mTICI, modified Thrombolysis in Cerebral Infarction; eTICI, expanded Treatment in Cerebral Ischemia.

**Figure 2:**
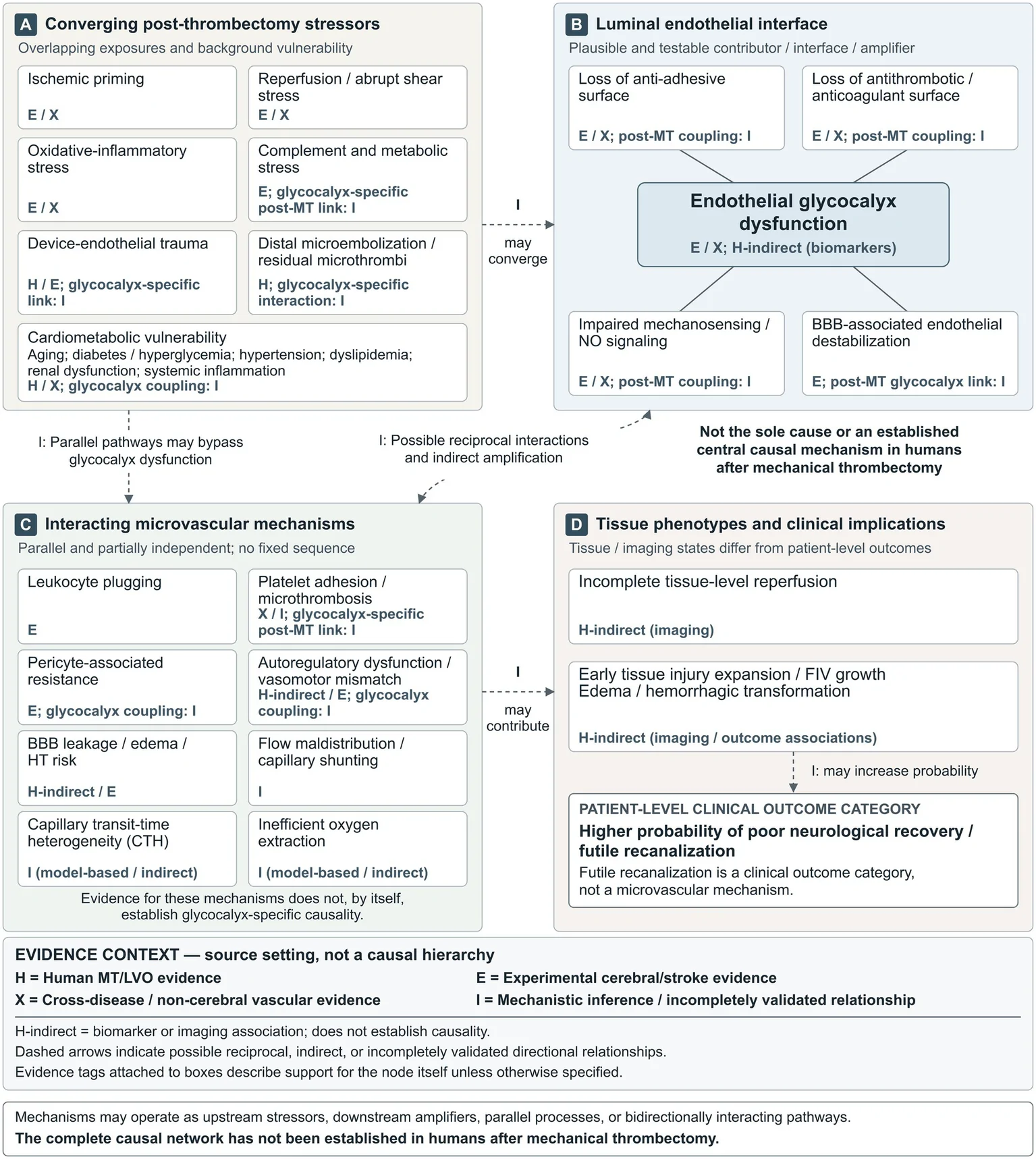
Conceptual network linking endothelial glycocalyx dysfunction to post-thrombectomy microvascular reperfusion failure. Evidence-context tags denote source setting and evidence directness, not a causal hierarchy: H, human MT/LVO evidence; E, experimental cerebral/stroke evidence; X, cross-disease/non-cerebral vascular evidence; I, mechanistic inference or an incompletely validated relationship; H-indirect, human biomarker or imaging association that does not establish causality. The figure is a network model rather than a confirmed linear causal pathway. **(A)** Summarizes converging post-thrombectomy stressors and background cardiometabolic vulnerability; **(B)** Positions endothelial glycocalyx dysfunction as a plausible and testable luminal endothelial interface/amplifier; **(C)** Summarizes interacting microvascular mechanisms that may operate in parallel, reciprocally, or independently; and **(D)** separates tissue/imaging phenotypes from patient-level clinical outcome. Dashed arrows indicate possible reciprocal, indirect, or incompletely validated directional relationships. The complete causal network has not been established in humans after mechanical thrombectomy, and evidence for individual microvascular mechanisms does not by itself establish glycocalyx-specific causality. Futile recanalization is a patient-level clinical outcome category, not a microvascular mechanism. BBB, blood–brain barrier; CTH, capillary transit-time heterogeneity; HT, hemorrhagic transformation; LVO, large-vessel occlusion; MT, mechanical thrombectomy.

Priority was given to five evidence contexts: (1) direct human evidence obtained in LVO/EVT or MT populations; (2) indirect human biomarker or imaging evidence relevant to the proposed pathway; (3) experimental cerebral ischemia or stroke evidence; (4) non-cerebral or cross-disease vascular evidence used to support endothelial or glycocalyx biology; and (5) hypothesis-generating mechanistic inference. These categories describe evidence context and directness; they are not a formal GRADE-type hierarchy. Where a specific mechanistic statement could be supported by an original study, primary evidence was prioritized over systematic reviews or meta-analyses, while secondary evidence was retained for incidence estimates, heterogeneity, or broader synthesis.

The final reference set contains 53 publications, including 38 primary clinical, experimental, or modeling reports and 15 systematic reviews, meta-analyses, or narrative/mechanistic reviews. Because the original search was performed as a narrative review and record-level deduplication and exclusion logs were not prospectively retained, a PRISMA-style denominator of all records screened cannot be reconstructed reliably and is therefore not reported. To improve transparency without overstating methodological systematicity, the revised manuscript specifies inclusion/exclusion considerations, provides the final included-reference count, documents representative search strategies, and summarizes design characteristics of all 38 primary clinical, experimental, and modeling reports in Supplementary Table S3. Reports were excluded from the core synthesis when they were not mechanistically relevant to the post-recanalization problem, did not address endothelial/glycocalyx biology or a competing microvascular mechanism, or duplicated higher-quality evidence without adding clinically or mechanistically distinct information.

A recent 2026 review of peripheral vascular disease was incorporated only as cross-disease vascular context for systemic endothelial dysfunction, nitric-oxide impairment, oxidative stress, inflammation, thrombosis, and cardiometabolic risk (11). It is not treated as direct evidence of cerebral glycocalyx injury after MT.

A 2026 narrative review published within the search window also focused on endothelial glycocalyx and inflammatory responses in patients with acute ischemic stroke treated with mechanical thrombectomy (12). The present review complements that literature by explicitly separating evidence contexts, distinguishing IMR/no-reflow from patient-level outcome categories, comparing competing microvascular mechanisms, and proposing a tiered biomarker-imaging framework for hypothesis testing.

## Definitions and conceptual hierarchy

Recanalization refers to reopening of the previously occluded parent artery. Angiographic reperfusion describes downstream contrast filling, generally graded by mTICI/eTICI. Tissue-level reperfusion addresses whether parenchymal blood flow and oxygen delivery are sufficient for threatened tissue to survive. Microvascular reperfusion is more granular and encompasses arteriolar, capillary, and venular patency, rheology, tone, permeability, and flow distribution. IMR is used here as a broad clinical/imaging concept for persistent or abnormal downstream microvascular perfusion after macrovessel recanalization. No-reflow is used more narrowly for persistent failure of microvascular/capillary reperfusion despite restoration of upstream patency, particularly in experimental or specifically operationalized clinical settings. Their operational definitions vary by imaging modality, threshold, anatomical scale, and timing (3–5, 13, 14).

Large-vessel reocclusion is a separate event: a previously reopened parent vessel becomes occluded again. It should not be conflated with IMR/no-reflow. Likewise, futile recanalization is a patient-level clinical outcome category, usually defined by poor functional outcome despite technically successful recanalization/reperfusion, and is influenced by age, baseline infarct core, collaterals, stroke severity, premorbid status, infection, rehabilitation, systemic complications, edema, HT, and comorbidity (2). Figure 1 summarizes these distinctions.

## The endothelial glycocalyx as a blood-facing BBB/NVU interface

The endothelial glycocalyx is a carbohydrate-rich luminal layer containing heparan sulfate, hyaluronan, chondroitin sulfate, syndecans, glypicans, mucin-domain glycoproteins, sialylated glycoproteins, and plasma-derived proteins (7–10). Its composition and thickness vary by vascular bed, flow state, inflammation, age, and disease context. In the brain, this layer operates within the specialized BBB and NVU, where endothelial cells, pericytes, basement membrane, astrocytic endfeet, microglia, neurons, and extracellular matrix jointly regulate barrier function and neurovascular coupling (9, 10, 15, 16). The glycocalyx is therefore related to, but is not synonymous with, either the BBB or the NVU.

An intact glycocalyx limits direct blood-cell contact with the endothelial membrane, supports antithrombotic and anticoagulant surface properties, contributes to permeability control, and participates in shear-dependent nitric-oxide (NO) signaling (6–8). Experimental cerebral ischemia work links glycocalyx integrity to suppression of endothelial transcytosis and preservation of BBB function (9), while recent brain-aging research shows that glycocalyx dysregulation and mucin-domain glycoproteins can influence BBB stability (10). These findings strengthen the anatomical rationale for a luminal-surface hypothesis, but they do not establish that glycocalyx disruption causes post-MT no-reflow in humans (Table 1).

**Table 1:** Conceptual hierarchy of post-thrombectomy reperfusion failure.

Term	Definition	Typical measurement	Mechanistic meaning	Clinical limitation
Recanalization	Reopening of the occluded parent artery.	Digital subtraction angiography (DSA), computed tomography angiography (CTA), magnetic resonance angiography (MRA); component of mTICI/eTICI assessment.	Restores proximal access for blood flow.	Does not prove parenchymal perfusion, oxygen delivery, or tissue survival.
Large-vessel reocclusion	Post-procedural re-occlusion of a previously reopened parent artery.	Repeat DSA, CTA, or MRA demonstrating renewed parent-vessel occlusion.	Loss of restored macrovessel patency; distinct from peri-procedural microvascular reperfusion failure/no-reflow.	Cannot be inferred from poor clinical outcome or downstream perfusion abnormality alone; requires repeat vascular imaging for confirmation.
Angiographic reperfusion	Contrast filling of the downstream vascular territory after treatment.	mTICI/eTICI grade on DSA.	Describes macroscopic flow restoration.	Insensitive to capillary patency, oxygen extraction, BBB leakage, and delayed infarct growth.
Tissue-level reperfusion	Adequate parenchymal perfusion and oxygen delivery to threatened tissue.	Computed tomography perfusion (CTP), magnetic resonance perfusion (MRP), arterial spin labeling (ASL), tissue outcome.	Intermediate layer between technical success and tissue salvage.	May include hypoperfusion, hyperperfusion, or maldistributed flow.
Microvascular reperfusion	Functional arteriolar, capillary, and venular flow with preserved rheology, tone, and barrier function.	DSA-PCC, CTP/MRP/ASL, BOLD-CVR, experimental microscopy.	Reflects downstream vascular-network function.	Difficult to measure directly in humans.
IMR	Broad clinical/imaging state of persistent or abnormal microvascular perfusion after macrovessel recanalization.	Post-MT perfusion imaging; angiographic flow metrics.	Can include persistent hypoperfusion or other downstream flow abnormalities.	Definitions and thresholds vary across modalities and time windows.
No-reflow	Persistent failure of microvascular/capillary reperfusion despite upstream recanalization.	Experimental capillary non-filling; specifically defined clinical imaging phenotypes.	May involve obstruction, constriction, edema, or rheologic failure.	Not completely synonymous with all IMR phenotypes.
Futile recanalization	Poor patient-level clinical outcome despite technically successful recanalization/reperfusion.	Commonly a 90-day modified Rankin Scale (mRS) threshold.	Clinical outcome category.	Multifactorial and should not be used as a stand-alone mechanistic endpoint.

## Cardiometabolic vulnerability and pre-existing endothelial-surface resilience

In this review, ‘cardiometabolic vulnerability’ denotes a host context in which chronic metabolic, inflammatory, renal, and vascular stress may reduce baseline endothelial-surface resilience before the ischemic event. It is a conceptual modifier rather than a diagnosis. The evidence context for this section is predominantly cross-disease/non-cerebral vascular evidence (6, 11), supplemented by experimental cerebral/brain-aging evidence (10) and indirect human clinical associations with post-EVT outcomes (2, 17, 18); direct human post-MT evidence linking these cardiometabolic states to cerebral glycocalyx injury is currently lacking (Table 2).

**Table 2:** Competing and interacting mechanisms of post-thrombectomy microvascular failure.

Mechanism	Potential relationship to glycocalyx dysfunction	Representative evidence context	Potential readout	Key limitation
Distal embolization	Upstream, parallel, or independent; can sustain local low flow and endothelial stress.	Human procedural primary (24) + experimental	DSA/CTA; distal occlusion; perfusion defects	Not glycocalyx-specific; can directly cause perfusion failure.
Microthrombosis	Potential downstream amplifier, parallel pathway, or bidirectional interaction.	Experimental primary (26); indirect human	Coagulation/fibrinolysis markers; perfusion imaging	Multiple drivers including thrombin, stasis, emboli, inflammation.
Leukocyte plugging	Potential downstream amplifier after loss of anti-adhesive surface.	Animal no-reflow primary (25); indirect human	Leukocyte/endothelial activation markers; experimental microscopy	No direct human proof that glycocalyx loss is the upstream trigger.
Pericyte contraction	Parallel/interacting pathway via NO, oxidative stress, edema.	Animal/ex vivo primary (30, 31)	Experimental microscopy; perfusion	Direct glycocalyx-pericyte coupling after MT untested.
Endothelial swelling	Parallel or interacting endothelial injury mechanism.	Experimental/indirect clinical; broader no-reflow synthesis (5)	Perfusion imaging; pathology	Hard to separate from edema and other endothelial injury.
BBB disruption/edema/HT	Potential downstream and parallel pathway.	Experimental cerebral (9, 16) + human MT/EVT primary (28, 29)	Permeability imaging; edema; HT	Multifactorial and not glycocalyx-specific.
Complement activation	Upstream/parallel inflammatory stressor.	Experimental cerebral primary (20) + cross-disease	C3a/C5a; inflammatory panels	Temporal complexity; pleiotropic effects.
Impaired autoregulation/vasomotor mismatch	Parallel/interacting; potentially amplified by impaired mechanosensing.	Human imaging primary (27) + mechanistic inference	BOLD-CVR; CTP/MRP/ASL	Not specific to luminal-surface injury.
Glycocalyx dysfunction	Proposed interface/amplifier linking blood-facing stress to adhesion, coagulation, mechanosensing, and barrier biology.	Human biomarker primary (38–43) + experimental/cross-disease	Serial compartment-annotated shedding markers + imaging	No direct in-vivo cerebral glycocalyx visualization immediately after human MT.

Cross-disease/systemic vascular studies support associations of diabetes and hyperglycemia with oxidative and inflammatory stress and impaired endothelial NO bioavailability, and of hypertension and dyslipidemia with altered endothelial mechanotransduction, abnormal hemodynamic stress, and inflammatory activation (6, 11). Renal dysfunction may influence systemic endothelial homeostasis, inflammation, and clearance of circulating glycocalyx-related biomarkers, but this relationship has not been validated as a cerebral post-MT mechanism. Experimental and translational brain evidence supports aging-related alterations in glycocalyx-associated BBB biology (10). Indirect clinical literature links cardiometabolic risk states to unfavorable post-EVT outcomes, but not specifically through glycocalyx injury (2, 17, 18). These factors may also worsen stroke outcome through glycocalyx-independent pathways and should therefore be modeled as confounders, effect modifiers, and biological background conditions rather than assumed to operate through a confirmed cerebral glycocalyx pathway after MT.

The proposed two-stage model is therefore hypothesis-generating: pre-existing cardiometabolic vulnerability may lower endothelial/glycocalyx reserve, after which acute ischemia, reperfusion, and mechanical stress may produce a larger or more persistent luminal-surface disturbance. This model is supported mainly by cross-disease/non-cerebral vascular and experimental evidence and should not be interpreted as direct proof of cerebral glycocalyx injury after MT.

## Triggers of glycocalyx dysfunction in the setting of MT

### Ischemic priming

During arterial occlusion, endothelial hypoxia, ATP depletion, acidosis, ionic dysregulation, low shear, and inflammatory signaling may weaken endothelial-surface homeostasis before the artery is reopened (6, 9, 17). Reperfusion may therefore act on an already vulnerable surface (Table 3).

**Table 3:** Evidence-context map for the endothelial glycocalyx hypothesis.

Evidence domain	Representative evidence	Refs	Interpretation	Evidence context/limitation
Brain glycocalyx and BBB/NVU biology	BBB glycocalyx, ischemic-stroke models, mucin-domain glycoprotein work	(9, 10, 15, 16)	Supports a blood-facing BBB-associated surface.	Experimental cerebral/brain-disease evidence; not direct human MT causality.
Ischemia–reperfusion glycocalyx degradation	Organ I/R literature and stroke BBB models	(6, 9, 17, 18)	Shows vulnerability of luminal surface to reperfusion stress.	Mostly experimental or non-cerebral; pathways not glycocalyx-specific.
Neutrophil plugging	Mouse no-reflow after recanalization	(25)	Provides a downstream mechanism glycocalyx loss could amplify.	Animal evidence; does not establish glycocalyx as upstream driver in humans.
Pericyte contraction	Rodent/ex vivo persistent capillary constriction	(30, 31)	Potential interaction with endothelial NO/edema pathways.	Animal evidence; direct coupling untested.
CTH and oxygen extraction	Modeling and clinical perfusion work	(27, 32–34)	Defines a hemodynamic phenotype potentially amplified by heterogeneous luminal injury.	Mechanistic inference/indirect clinical evidence.
Intracranial vs. peripheral syndecan-1	Local distal and peripheral biomarker heterogeneity	(38)	Supports compartment-specific sampling.	Relatively direct human biomarker evidence; small cohort, no direct visualization.
Peripheral biomarker dynamics	Serial EVT/MT and thrombolysis cohorts	(39–43)	Suggests time-dependent endothelial/glycocalyx-related signals.	Indirect human biomarker evidence; systemic and timing-dependent.
Post-MT imaging phenotypes	BOLD-CVR, CTP, DSA-PCC, serial perfusion MRI, FIV/edema/HT	(13, 14, 27–29, 35–37)	Defines intermediate and time-varying reperfusion/tissue states for hypothesis testing.	Indirect human evidence; imaging patterns not glycocalyx-specific.
Post-EVT microcirculatory rescue	CHOICE, PEARL, intra-arterial tenecteplase	(44–49)	Shows residual downstream dysfunction may be therapeutically actionable.	Direct human rescue concept; not direct glycocalyx evidence.
Putative glycocalyx-directed therapy	Sulodexide/heparanase/GAG restoration	(50–53)	Mechanistically relevant intervention class.	Predominantly non-brain/non-MT or preclinical.
Cardiometabolic vulnerability	Diabetes, hyperglycemia, hypertension, dyslipidemia, renal/systemic endothelial dysfunction	(2, 6, 10, 11, 17, 18)	Provides host-risk context for reduced endothelial-surface resilience.	Indirect clinical context (2, 17, 18); experimental brain-aging evidence (10); cross-disease/non-cerebral vascular context (6, 11). Not proof of cerebral glycocalyx causality after MT.

### Reperfusion-associated oxidative and inflammatory stress

Restoration of oxygen and substrate also produces abrupt shear changes, reactive oxygen species, neutrophil recruitment, thrombin generation, and cytokine signaling that may promote enzymatic shedding of glycocalyx components (6, 17–19).

Complement and metabolic stress. Complement activation contributes to cerebral ischemia–reperfusion injury in experimental models (19, 20), while experimental MT models implicate succinate-associated metabolic injury (18, 19). These processes are not glycocalyx-specific and may damage multiple NVU compartments (Table 4).

**Table 4:** Imaging and tissue endpoints for future mechanistic studies.

Endpoint	Window	Biological meaning	Advantages	Limitations/future use
DSA-PCC	Immediately post-recanalization	Angio-suite flow delay and parametric hemodynamic state	Immediate and procedure-linked	Requires standardization; candidate core endpoint for immediate IMR.
CTP	Early post-MT, ideally 1–6 h; up to 72 h	Hypoperfusion, hyperperfusion, perfusion delay	Clinically familiar	Threshold/protocol heterogeneity; pair with biomarkers and 24-h tissue outcome.
MRP	Early post-MT; repeat at 24–48 h when feasible in mechanistic cohorts	Parenchymal perfusion and transit delay	Rich tissue characterization with DWI/FLAIR	Logistical barriers; useful for dynamic no-reflow trajectories and secondary mechanistic endpoints.
ASL	Early and follow-up	Noncontrast CBF and arterial-transit effects	Repeatable; no contrast	Transit artifacts; useful exploratory serial trajectory.
BBB permeability imaging	Early post-MT and ~24 h	Barrier leakage and edema/HT risk	Mechanistically close to endothelial injury	Limited availability; selected mechanistic cohorts.
BOLD-CVR	Early/subacute	Vasomotor reserve and steal physiology	Captures autoregulatory dysfunction	Less available acutely; supportive/exploratory endpoint.
FIV/infarct growth	12–96 h; ~24 h preferred in early trials	Tissue injury expansion	Closer to mechanism than 90-day mRS	Downstream and multifactorial; secondary mechanistic endpoint.
Edema/HT/sICH	12–96 h	Barrier failure, mass effect, safety state	Clinically important	Multifactorial; safety and barrier-related endpoint.
NIHSS change	24 h, 7 d, discharge	Early neurological response	Clinically meaningful and temporally closer	Affected by lesion eloquence, sedation, edema, complications.
90-day mRS	90 d	Patient-level functional outcome	Standard efficacy endpoint	Use as clinical endpoint, not sole mechanistic endpoint.

### Device-endothelial trauma and altered shear

Stent retrievers, aspiration catheters, repeated passes, clot-device interaction, and rapid flow restoration can perturb the vessel wall and endothelium (21–23). Direct visualization of cerebral glycocalyx damage from thrombectomy devices in patients remains unavailable.

### Distal embolization and residual microthrombotic burden

Distal clot migration or residual microemboli can create patchy low-flow territories and sustain thromboinflammation (5, 24). These processes may occur upstream of, downstream of, or independently from glycocalyx dysfunction.

### Host vulnerability

Age, diabetes, acute hyperglycemia, hypertension, dyslipidemia, renal dysfunction, infection, systemic inflammation, endothelial dysfunction, and peri-procedural blood-pressure variability may reduce resilience or alter recovery (2, 6, 10, 11).

## Glycocalyx dysfunction within a network of microvascular failure

Post-thrombectomy microvascular failure is unlikely to be explained by a single linear cascade. Distal embolization, microthrombosis, leukocyte plugging, pericyte contraction, endothelial swelling, BBB disruption, complement activation, impaired autoregulation, edema, and host vulnerability may occur upstream of glycocalyx dysfunction, downstream of it, in parallel, or independently. A network model is therefore more credible than a single-cause model. In this framework, glycocalyx dysfunction is proposed as a blood-facing interface or amplifier that can make established no-reflow mechanisms more likely, persistent, or spatially heterogeneous.

### Loss of anti-adhesive function and leukocyte plugging

An intact glycocalyx separates circulating leukocytes from endothelial adhesion platforms. Thinning or shedding could facilitate rolling, adhesion, and capillary stalling (6–8). In a mouse recanalization model, neutrophils obstructed a substantial fraction of capillaries after upstream flow restoration, supporting leukocyte plugging as a no-reflow mechanism (25). That experiment does not establish glycocalyx shedding as the initiating event in human MT.

Platelet adhesion, microthrombosis, and antithrombotic-surface loss. Glycocalyx disruption may expose proadhesive/procoagulant surfaces and reduce local antithrombotic capacity (6–8). Primary experimental stroke work demonstrates capillary fibrin deposition and stacked erythrocytes after recanalization/thrombolysis, supporting a direct microvascular thrombotic component of no-reflow (26). Broader no-reflow syntheses likewise recognize microthrombi and fibrin-platelet aggregates (5), but the fraction attributable specifically to cerebral glycocalyx dysfunction remains unknown.

Impaired mechanosensing and NO-dependent vasomotor mismatch. Glycocalyx-dependent mechanotransduction links shear stress to endothelial nitric oxide (NO) production (7, 8). If the surface is damaged, sensing of restored flow may become heterogeneous, potentially contributing to vasomotor mismatch or impaired autoregulation. Blood oxygenation level-dependent cerebrovascular reactivity (BOLD-CVR) steal after successful EVT supports persistent hemodynamic dysfunction, but not glycocalyx causality (27).

BBB destabilization, edema, and HT. Experimental stroke studies show that glycocalyx disruption can increase endothelial transcytosis and BBB permeability (9, 16). Human MT/EVT imaging studies also show that BBB disruption or increased permeability is associated with hemorrhagic transformation and adverse tissue states (28, 29), although these observations are not glycocalyx-specific. Glycocalyx dysfunction is therefore a plausible participant in barrier failure, but current human evidence does not establish independent glycocalyx causation of edema or HT after MT.

### Pericyte-associated resistance

Pericyte-mediated capillary constriction is well supported experimentally after cerebral ischemia (30, 31). Glycocalyx dysfunction may interact through NO signaling, endothelial stress, edema, or leukocyte stalling, but direct post-MT glycocalyx-pericyte coupling has not been demonstrated.

### Capillary transit-time heterogeneity (CTH), shunting, and oxygen extraction

Theoretical and imaging work indicates that heterogeneous capillary transit can impair oxygen extraction even when total cerebral blood flow is not uniformly reduced (32–34). Patchy adhesion, microthrombosis, vasomotor mismatch, and barrier leakage could contribute, but perfusion-metabolic validation is required in MT populations.

## Incomplete tissue-level reperfusion as an intermediate phenotype

Incomplete tissue-level reperfusion is best treated as an intermediate tissue/imaging phenotype between technical success and clinical outcome. It may include persistent hypoperfusion, regional hyperperfusion, capillary flow maldistribution, CTH, impaired oxygen extraction, BBB leakage, edema, and evolving infarct growth (3, 4). These abnormalities can raise the risk of poor recovery but are not equivalent to futile recanalization.

Persistent hypoperfusion may reflect IMR, residual or distal emboli, diffuse flow limitation, vasomotor resistance, or edema-related compression. Hyperperfusion may indicate autoregulatory failure and barrier vulnerability. Maldistributed capillary flow may reduce oxygen extraction efficiency. Recent prospective human imaging studies further emphasize both mechanistic heterogeneity and temporal dynamics: early post-EVT perfusion deficits were often attributable to residual distal embolic or macrovascular abnormalities rather than visually defined microvascular hypoperfusion, whereas serial perfusion MRI after eTICI 2c-3 reperfusion demonstrated persistent, resolving, progressive, and newly developing no-reflow patterns across early and 24–48 h assessments (13, 14). Follow-up imaging measures such as final infarct volume (FIV), infarct growth, edema, and HT are therefore more temporally proximate to peri-procedural vascular injury than 90-day mRS alone (13, 14, 27, 35–37). Mechanistic studies should pair patient-level outcomes with early and, where feasible, serial imaging endpoints rather than infer microvascular causality from disability alone.

## Biomarker evidence: time, compartment, and interpretation

Most human work uses circulating shedding products, particularly syndecan-1, heparan sulfate, and hyaluronan. Their practical advantage is serial accessibility; their central limitation is lack of cerebral specificity. Peripheral concentrations may reflect lung, kidney, liver, gut, systemic endothelium, vascular instrumentation, clearance, or systemic inflammation in addition to cerebral endothelial biology.

The closest human evidence comes from LVO cohorts with paired intracranial and peripheral sampling. Intracranial and peripheral syndecan-1 show spatiotemporal heterogeneity, supporting the principle that local distal-occlusion signals and peripheral venous concentrations should not be assumed to be synchronized (38). Longitudinal EVT/MT cohorts also demonstrate that syndecan-1 behavior depends on sampling time and clinical context (39–41). A thrombolysis-treated AIS cohort found higher plasma syndecan-1 associated with unfavorable outcome (42), whereas an MT cohort reported that lower postoperative-day-1 syndecan-1 was associated with worse prognosis (40). These apparently divergent findings illustrate why concentration direction at a single time point cannot be translated directly into ‘more’ or ‘less’ cerebral glycocalyx injury.

Additional prospective 2026 data reinforce the need for time- and treatment-context interpretation. Babić et al. measured syndecan-1, heparan sulfate, hyaluronan, and IL-18 before recanalization and at 24 and 48 h in patients treated with intravenous thrombolysis, mechanical thrombectomy, or combination therapy (43). The study identified treatment-group differences across serial measurements, while syndecan-1 was not directly associated with NIHSS or mRS. These findings do not establish cerebral glycocalyx injury, but they further argue against interpreting a single circulating concentration as a monotonic measure of brain-specific glycocalyx loss.

An early increase may be compatible with acute ectodomain shedding and reperfusion-associated washout; a later decrease could reflect depletion of releasable surface material, altered clearance, compartment redistribution, repair kinetics, or differences in systemic contribution. Conversely, later increases could reflect continuing endothelial injury or secondary inflammatory responses. These interpretations remain biologically plausible hypotheses rather than validated kinetic rules and have not been prospectively validated as temporal markers of cerebral glycocalyx injury in human MT cohorts. Renal function, diabetes, hypertension, infection, systemic inflammation, anesthesia, contrast exposure, heparin, intravenous thrombolysis, blood-pressure treatment, number of device passes, reperfusion grade, and sampling time can all influence interpretation.

Accordingly, peripheral syndecan-1 should be described as an imperfect systemic shadow of endothelial/glycocalyx injury, not a real-time quantitative mirror of the cerebral microvascular glycocalyx. Future studies should prioritize trajectories over isolated values, annotate the sampling compartment, and pair biomarker kinetics with temporally aligned perfusion and BBB imaging. Candidate blood biomarkers, suggested sampling windows, and interpretation limitations are summarized in Supplementary Table S1.

## Therapeutic implications

### Clinically evaluated microcirculatory rescue after angiographic reperfusion

Adjunctive intra-arterial thrombolysis is the most clinically advanced example of post-recanalization microcirculatory rescue. CHOICE, a phase 2b randomized placebo-controlled trial, suggested a higher frequency of excellent outcome with intra-arterial alteplase after successful thrombectomy, although the trial was stopped early and required replication (44). PEARL subsequently reported a higher proportion of 90-day modified Rankin Scale (mRS) 0–1 with intra-arterial alteplase after successful endovascular reperfusion in selected patients (45). Phase 1b/2a intra-arterial tenecteplase data support dose selection and safety for further study but do not yet establish definitive clinical efficacy (46). Meta-analyses provide broader synthesis of this treatment concept (47–49). These studies support therapeutic actionability of the post-EVT microcirculation; they do not establish glycocalyx repair as the mechanism of benefit.

### Mechanism-adjacent endothelial or reperfusion protection

Strategies directed at oxidative stress, complement activation, metabolic stress, endothelial inflammation, BBB dysfunction, edema, glucose control, blood-pressure variability, and systemic inflammatory burden may indirectly influence the luminal endothelial surface (11, 17–19). Their potential effects are pleiotropic and should not be labeled glycocalyx-specific. In translational trials, cardiometabolic phenotype should be treated both as a biological modifier and as a prespecified adjustment variable.

### Putative glycocalyx-directed therapies

Sulodexide, heparanase-related strategies, glycosaminoglycan restoration, and preservation of hyaluronan/heparan sulfate are conceptually attractive (50–53). However, supporting data derive largely from diabetes, sepsis, non-cerebral vascular injury, animal models, or endothelial-cell systems. Acute post-MT stroke differs in BBB vulnerability, bleeding risk, antithrombotic exposure, infarct biology, and therapeutic time window. These approaches should therefore be described as experimental, cross-disease, or trial-enabling candidates rather than validated post-thrombectomy glycocalyx treatments.

## Research agenda: a feasible tiered prospective framework

The next phase of research should be designed to support, refine, or reject the glycocalyx hypothesis. Five principles are central: precisely define the target reperfusion phenotype; sample biomarkers serially and, where feasible, by compartment; align biomarker and imaging acquisition closely enough to test temporal ordering; model mediation rather than simple association; and prespecify cardiometabolic vulnerability as both a confounder and potential effect modifier. An operationally feasible prospective cohort design is outlined in Box 1.

Box 1 **Proposed prospective cohort after MT**

**Population.** Adults with anterior-circulation LVO treated with MT who achieve near-complete or complete angiographic reperfusion, preferably mTICI/eTICI 2c-3, with sensitivity analyses restricted to eTICI 3. Record baseline core, collateral status, stroke severity, procedural variables, diabetes, admission glucose, HbA1c when available, hypertension, dyslipidemia, renal function, systemic inflammation, and other relevant vascular comorbidity indicators.

**Core sampling.** Pre-MT peripheral venous blood; immediate post-recanalization blood; 1–6 h; and 24 h. Local arterial blood proximal/distal to the occlusion should be collected only where technically and ethically feasible.

Core biomarkers/endpoints. Syndecan-1 trajectory as the principal glycocalyx-related biomarker; admission glucose/HbA1c, creatinine/eGFR and inflammatory indices; a prespecified immediate or early perfusion endpoint [DSA-PCC or computed tomography perfusion (CTP), according to site capability]; 24-h final infarct volume (FIV)/infarct growth, edema and hemorrhagic transformation (HT); 24-h National Institutes of Health Stroke Scale (NIHSS) and 90-day mRS.

**Secondary mechanistic markers.** Heparan sulfate, hyaluronan, thrombomodulin, von Willebrand factor, endothelial activation markers, MMP-9, C3a/C5a, coagulation-fibrinolysis markers, and leukocyte indices.

Exploratory markers/endpoints. Broader cytokine panels, advanced BBB permeability imaging, magnetic resonance perfusion (MRP), arterial spin labeling (ASL), BOLD-CVR, and center-specific omics or endothelial assays.

**Primary mechanistic endpoint.** A prespecified imaging-defined phenotype of incomplete microvascular/tissue-level reperfusion. For example, in centers using DSA-PCC, the primary endpoint could be abnormal parenchymal contrast-transit delay measured immediately after final angiographic reperfusion; in centers using CTP, it could be residual hypoperfusion volume measured at 1–6 h after MT. The specific perfusion metric, acquisition protocol, anatomical definition, and abnormality threshold should be prespecified prospectively before enrollment or outcome analysis. Modality-specific definitions should be analyzed separately unless a harmonized cross-modality definition has been validated.

**Analysis.** Test a prespecified pathway from biomarker dynamics to imaging-defined IMR to tissue injury expansion and clinical outcome; adjust for core volume, collateral status, procedural variables, systemic inflammation, renal function, cardiometabolic factors, and treatment exposures; test interactions between metabolic vulnerability and biomarker/imaging relationships.

**Key limitations.** Observational design, biomarker nonspecificity, sampling feasibility, timing mismatch, confounding, and absence of direct in-vivo cerebral glycocalyx imaging.

## Limitations of the glycocalyx hypothesis

First, cerebral microvascular glycocalyx disruption has not been directly visualized in patients immediately after MT. Much of the mechanistic rationale comes from animal models, ex vivo systems, non-cerebral vascular beds, or indirect human biomarker studies. Second, peripheral syndecan-1, heparan sulfate, and hyaluronan are not brain-specific and are affected by systemic endothelial injury, clearance, comorbidity, and treatment exposure. Third, temporal ordering between imaging-defined IMR/no-reflow and biomarker shedding has not been rigorously established. Fourth, distal embolization, pericyte contraction, microthrombosis, endothelial swelling, complement activation, BBB injury, edema, inflammation, and host vulnerability can occur upstream of, downstream of, in parallel with, or independently from glycocalyx dysfunction. Fifth, candidate glycocalyx-protective therapies are supported mainly by non-brain/non-MT or preclinical evidence. Sixth, the narrative search did not prospectively retain record-level screening counts and therefore cannot support PRISMA-style completeness claims. These limitations define the experiments and clinical designs required to test the model rather than diminishing the value of the hypothesis as a research framework.

## Discussion and conclusion

Endothelial glycocalyx dysfunction offers a plausible way to organize several blood-facing processes that may contribute to the gap between successful macrovessel recanalization and effective tissue-level reperfusion. Its biological appeal lies in its location at the interface of shear stress, inflammation, coagulation, leukocyte/platelet interaction, endothelial signaling, BBB stability, and cardiometabolic vulnerability. However, the glycocalyx is not established as the primary cause of human post-MT no-reflow, and circulating shedding markers are indirect and nonspecific.

The most productive near-term strategy is therefore not empiric glycocalyx-targeted treatment, but rigorous phenotype definition: serial, compartment-annotated biomarker trajectories integrated with temporally aligned perfusion/barrier imaging, tissue injury endpoints, and explicit modeling of competing mechanisms and host vulnerability. If glycocalyx-related biomarker dynamics reproducibly track imaging-defined microvascular reperfusion failure and mediate subsequent tissue injury, the framework could support risk stratification and mechanism-informed trials. Until then, its principal translational value is as a testable interface linking host vascular vulnerability, reperfusion-associated stress, and heterogeneous downstream microvascular dysfunction after technically successful thrombectomy.
