# Supplementary material for: Endothelial glycocalyx dysfunction in the setting of mechanical thrombectomy: a conceptual link between cardiometabolic vulnerability and microvascular reperfusion failure
Source: Front Neurol. 2026 Oct 21;17:1919445. doi: 10.3389/fneur.2026.1919445 (PMC13732575; doi:10.3389/fneur.2026.1919445)
Supplement: Supplementary file 1 [file Table_1.docx]

Supplementary Table S1. Candidate blood biomarkers and cardiometabolic laboratory covariates for future studies.

| **Biomarker** | **Biological meaning** | **Suggested sampling** | **Evidence / representative refs** | **Key limitations** | **Recommended role** |
| --- | --- | --- | --- | --- | --- |
| Syndecan-1 | Proteoglycan ectodomain shedding; endothelial/glycocalyx-related signal. | Pre-MT; local arterial if feasible; immediate; 1-6 h; 24 h; 72 h; 7 d. | Human LVO/EVT/MT: refs 38-43. | Not brain-specific; strongly timing-, compartment-, treatment-, and clearance-dependent. | Core serial biomarker; avoid single-time-point interpretation. |
| Heparan sulfate | Major glycocalyx glycosaminoglycan; possible heparanase-related degradation. | As for syndecan-1; annotate heparin exposure. | Human EVT/MT cohorts measured HS (39,43); broader glycocalyx biology (6-8). | Heparin/clearance/systemic endothelial injury confound interpretation. | Secondary panel marker; not stand-alone endpoint. |
| Hyaluronan | Glycocalyx matrix component and inflammation-associated ECM signal. | Baseline through 7 d. | Human EVT/MT cohorts measured HA (39,43); broader glycocalyx biology (6-8). | Highly nonspecific; inflammation and renal clearance influence levels. | Secondary marker interpreted with syndecan-1/HS and imaging. |
| Thrombomodulin | Endothelial injury and anticoagulant-surface disruption. | Immediate to 72 h. | General endothelial/glycocalyx anticoagulant-surface biology (6-8); no MT-specific validation in this review. | Not glycocalyx-specific. | Secondary endothelial injury marker with thrombotic/HT endpoints. |
| von Willebrand factor | Endothelial activation and platelet-adhesion biology. | Pre-MT to 72 h. | Endothelial activation/platelet-adhesion rationale (5-8); no glycocalyx-specific MT validation. | Age, blood group, inflammation, comorbidity. | Secondary covariate / endothelial activation marker. |
| MMP-9 / MMPs | Matrix degradation, BBB disruption, hemorrhage/edema risk. | 1-6 h; 24 h; 72 h. | Reperfusion/BBB-injury context (5,17); not luminal-surface-specific and not validated as a glycocalyx marker after MT. | Not glycocalyx-specific. | Secondary marker paired with BBB permeability/HT imaging. |
| C3a / C5a | Complement activation and inflammatory amplification. | Immediate-24 h; consider 72 h. | Cerebral complement/reperfusion evidence (19,20). | Complex temporal biology; not glycocalyx-specific. | Secondary multi-pathway inflammatory marker. |
| Inflammatory cytokines | Systemic/local inflammatory response. | Pre-MT to 7 d. | Reperfusion/inflammation literature (17,18); cross-disease vascular context (11); serial AIS recanalization biomarker data (43). | Low specificity; infection/systemic stress. | Adjustment/pathway-clustering variables. |
| ICAM-1 / VCAM-1 / E-selectin | Endothelial activation and leukocyte-adhesion signals. | Pre-MT to 72 h. | Mechanistically linked to leukocyte-endothelial interaction (6-8,25). | Assay variability and systemic endothelial contribution. | Secondary marker with leukocyte counts and imaging-defined IMR. |
| Coagulation-fibrinolysis panel | Microthrombotic and fibrinolytic milieu. | Pre-MT to 72 h. | No-reflow/microthrombosis and lytic-treatment context (5,44-49). | Clot burden, IVT, heparin, anticoagulants and systemic disease. | Covariates for microthrombosis and adjunct lytic studies. |
| Glucose/HbA1c/lipids/creatinine-eGFR | Cardiometabolic vulnerability, renal function, systemic context. | Baseline; glucose dynamic 24-72 h where relevant. | Indirect clinical/stroke context (2,17,18); experimental brain-aging evidence (10); general/cross-disease vascular context (6,11). | Not glycocalyx-specific; acute stress and treatment affect values. | Core covariates/stratifiers/effect modifiers. |

Supplementary Table S2. Representative reproducible database-specific search strategies for the narrative review.

| **Database** | Representative reproducible search example(s) | Coverage / update | **Selection approach** |
| --- | --- | --- | --- |
| PubMed/MEDLINE | Example query family: (("mechanical thrombectomy"[Title/Abstract] OR "endovascular therapy"[Title/Abstract] OR "endovascular treatment"[Title/Abstract]) AND ("no-reflow"[Title/Abstract] OR "impaired microvascular reperfusion"[Title/Abstract] OR "tissue reperfusion"[Title/Abstract] OR "futile recanalization"[Title/Abstract]) AND (glycocalyx[Title/Abstract] OR "syndecan-1"[Title/Abstract] OR "heparan sulfate"[Title/Abstract] OR hyaluronan[Title/Abstract] OR "blood-brain barrier"[Title/Abstract] OR pericyte*[Title/Abstract] OR leukocyte*[Title/Abstract] OR microthromb*[Title/Abstract] OR complement[Title/Abstract] OR perfusion[Title/Abstract])). Additional targeted searches paired MT/EVT terms with cardiometabolic-risk, imaging, or rescue terms. | Updated through 30 Apr 2026 | Targeted title/abstract review; primary studies prioritized for specific mechanistic claims. |
| Web of Science | TS=(("mechanical thrombectomy" OR "endovascular therapy" OR "endovascular treatment") AND ("no-reflow" OR "impaired microvascular reperfusion" OR "tissue reperfusion" OR "futile recanalization") AND (glycocalyx OR "syndecan-1" OR "heparan sulfate" OR hyaluronan OR "blood-brain barrier" OR pericyte* OR leukocyte* OR microthromb* OR complement OR perfusion)). Additional topic searches substituted cardiometabolic-risk, BBB/NVU, imaging, and microcirculatory-rescue terms as appropriate. | Updated through 30 Apr 2026 | Used to identify cross-disciplinary and citation-linked evidence. |
| Embase | (('mechanical thrombectomy':ti,ab OR 'endovascular therapy':ti,ab OR 'endovascular treatment':ti,ab) AND ('no-reflow':ti,ab OR 'impaired microvascular reperfusion':ti,ab OR 'tissue reperfusion':ti,ab OR 'futile recanalization':ti,ab) AND ('glycocalyx':ti,ab OR 'syndecan 1':ti,ab OR 'heparan sulfate':ti,ab OR hyaluronan:ti,ab OR 'blood brain barrier':ti,ab OR pericyte*:ti,ab OR leukocyte*:ti,ab OR microthromb*:ti,ab OR complement:ti,ab OR perfusion:ti,ab)) AND [english]/lim. Additional targeted searches used cardiometabolic, endothelial-injury, imaging, and rescue terms. | Updated through 30 Apr 2026 | Clinical and experimental studies screened for mechanistic relevance. |
| Google Scholar | Targeted phrase/citation searches, including: "mechanical thrombectomy" no-reflow; "mechanical thrombectomy" glycocalyx; "mechanical thrombectomy" "syndecan-1"; "DSA-PCC" microvascular failure; "serial perfusion MRI" thrombectomy no-reflow; and "intra-arterial thrombolysis" successful reperfusion. Citation chaining was used for key primary studies and reviews. | Updated through 30 Apr 2026 | Used to identify cited/related reports not captured by narrower database strings. |
| Reference-list screening | Backward/forward checking of key reviews and primary reports. | During manuscript preparation | Added studies if they contributed distinct mechanistic, biomarker, imaging, or therapeutic evidence. |

Search transparency note: The final narrative synthesis contains 53 publications (38 primary clinical/experimental/modeling reports and 15 secondary review/meta-analytic sources). Because record-level deduplication and exclusion logs were not prospectively retained during the original narrative search, exact screened/excluded denominators cannot be reconstructed reliably and are not reported. Supplementary Table S2 therefore provides reproducible representative database-specific search strings, the literature-coverage date, and selection logic rather than a PRISMA-style flow diagram.

Supplementary Table S3. Design characteristics of all 38 primary clinical, experimental, and modeling reports included in the narrative synthesis.

*For complex experimental or multimodal studies without a single study-wide denominator, the sample-size column reports the principal analytic cohort or representative per-experiment sample size; where a single total N is not meaningful, this is stated explicitly rather than summing heterogeneous experimental units.*

| **Study** | **Setting / design** | **Prospective / retrospective** | **Randomized / controlled** | **Primary / secondary analysis** | **Sample size / analytic unit** | **Main relevance** |
| --- | --- | --- | --- | --- | --- | --- |
| Barbagallo et al., 2025 (4) | IMPreST subanalysis after successful MT with serial multimodal perfusion/hemodynamic imaging | Prospective longitudinal observational cohort; prespecified subanalysis | Nonrandomized | Secondary analysis of prospectively collected cohort data | 18 analyzed (49 in parent IMPreST cohort) | Demonstrates heterogeneous post-MT hypo-, normalized, and hyperperfusion phenotypes. |
| Kutuzov et al., 2018 (16) | Mouse two-photon single-capillary BBB transport study with permeability modeling | Experimental; not applicable | Nonrandomized; within-study comparison across tracer sizes | Primary experimental analysis | Multiple mouse/capillary imaging experiments; no single study-wide N | Supports a luminal glycocalyx contribution to BBB barrier properties; not MT-specific. |
| Zhu et al., 2022 (9) | Mouse t-MCAO with serial BBB/glycocalyx assessment and glycocalyx manipulation | Experimental; longitudinal reperfusion time course | Controlled experimental groups; pharmacologic/enzymatic manipulations | Primary experimental analysis | Multiple mouse cohorts/time points; per-experiment n reported in source | Links glycocalyx integrity to endothelial transcytosis, BBB permeability, edema, and infarct injury. |
| Shi et al., 2025 (10) | Multiplatform ageing/disease study using mouse brain endothelium, glycocalyx perturbation, and human post-mortem tissue | Experimental/translational; not applicable | Controlled experimental comparisons; randomization not uniformly applicable across platforms | Primary experimental/translational analysis | Multiple cohorts; key glycocalyx assays commonly n=4-7 mice/group; human tissue cohort described in supplement | Establishes brain-specific glycocalyx dysregulation as relevant to BBB stability in ageing/disease; not post-MT evidence. |
| El Amki et al., 2020 (25) | Mouse thrombin MCA-occlusion/recanalization model with in-vivo capillary imaging and neutrophil depletion | Experimental; acute reperfusion study | Controlled treatment comparisons | Primary experimental analysis | Multiple mouse cohorts; per-experiment n reported in source | Strong animal evidence that neutrophil capillary plugging contributes to no-reflow after upstream recanalization. |
| Shrouder et al., 2024 (30) | Mouse experimental stroke with longitudinal in-vivo two-photon single-cell/pericyte imaging and molecular analyses | Experimental; longitudinal over 7 days | Controlled experimental comparisons | Primary experimental analysis | Multiple mouse imaging/molecular cohorts; no single study-wide N | Shows persistent pericyte dysfunction and capillary constriction as a post-ischemic no-reflow mechanism. |
| Korte et al., 2022 (31) | Rodent ex-vivo/in-vivo ischemia experiments with TMEM16A manipulation plus human tissue/genetic analyses | Experimental/translational; not applicable | Controlled pharmacologic/genetic experiments | Primary experimental/translational analysis | Multiple cohorts; key capillary-perfusion experiment: sham n=4, ischemia n=10, ischemia+Ani9 n=9 mice | Provides mechanistic evidence that pericyte TMEM16A activity amplifies constriction and reduces reperfusion. |
| Jespersen & Ostergaard, 2012 (32) | Biophysical modeling of CBF, capillary transit-time heterogeneity, oxygen extraction, and metabolism | Modeling study; not applicable | Not applicable | Primary modeling analysis | No participant cohort; mathematical model supported by published rat microvascular-flow data | Defines how heterogeneous capillary transit can reduce oxygen extraction despite restored flow. |
| Angleys et al., 2015 (33) | Extended flow-diffusion modeling of CTH and tissue oxygenation | Modeling study; not applicable | Not applicable | Primary modeling analysis | No participant cohort; mathematical simulations | Shows conditions in which increasing CBF may fail to improve oxygen delivery when CTH is high. |
| Engedal et al., 2018 (34) | Historic acute ischemic-stroke perfusion-MRI cohort with voxel-wise transit-time analysis plus computational modeling | Retrospective/historic clinical imaging cohort | Nonrandomized | Primary clinical imaging analysis with modeling | 126 acute ischemic stroke patients | Connects transit-time heterogeneity/homogenization with penumbral microvascular failure and tissue outcome. |
| Yin et al., 2022 (38) | Single-center longitudinal LVO biomarker cohort with intracranial + peripheral sampling | Longitudinal observational; prospective recruitment described | Nonrandomized; healthy comparator | Primary analysis | 24 LVO + 8 healthy | Compartment-specific syndecan-1 kinetics after successful reperfusion. |
| Liang et al., 2023 (39) | Single-center longitudinal EVT biomarker cohort | Observational longitudinal; prospectivity not explicitly labeled in the article | Nonrandomized; outcome-defined groups | Primary analysis | 80 analyzed (101 screened) | Serial syndecan-1/HA/HS and association with deterioration/mortality. |
| Xu et al., 2024 Acta (40) | Anterior-circulation LVO MT biomarker cohort | Observational; design timing not explicit in abstract | Nonrandomized | Primary analysis | 105 | Postoperative-day-1 syndecan-1 and 90-day prognosis. |
| Zhao et al., 2022 (42) | AIS intravenous-thrombolysis biomarker study | Observational cohort | Nonrandomized; healthy comparator | Primary analysis | 108 AIS + 47 healthy controls | Higher syndecan-1 associated with unfavorable outcome after rt-PA; non-MT-specific. |
| Xu et al., 2024 Front Neurol (41) | Anterior-circulation LVO MT cohort comparing IVT+MT versus direct MT | Retrospective | Nonrandomized; comparative | Primary analysis | 106 | Shows treatment-exposure effects on syndecan-1 dynamics and potential therapy-related confounding. |
| Teng et al., 2015 (21) | In-vitro live endothelial vessel platform with porcine thrombectomy validation | Experimental; not applicable | Controlled device comparisons; randomization not reported | Primary experimental analysis | Multiple in-vitro device-vessel experiments plus porcine validation; no single study-wide N reported | Demonstrates device-specific endothelial trauma and supports plausibility of thrombectomy-related luminal injury. |
| Donnerstag et al., 2026 (22) | Histomorphological study of tissue adherent to stentrievers and paired aspiration specimens after MT | Retrospective | Nonrandomized; within-patient specimen comparison | Primary analysis | 21 patients | Identifies vessel-wall tissue on thrombectomy devices, supporting procedural vessel-wall injury. |
| Kasab et al., 2021 (23) | Prospective high-resolution vessel-wall MRI within 24 h after MT | Prospective pilot cohort | Nonrandomized; target vessel compared with reference vessel | Primary analysis | 20 patients; 22 target vessels + 20 reference vessels | Shows post-MT arterial-wall enhancement consistent with vessel-wall/endothelial perturbation. |
| Mohammaden et al., 2020 (24) | Single-center anterior-circulation LVO MT cohort evaluating distal clot migration | Retrospective | Nonrandomized | Primary analysis | 65 patients (22 with distal clot migration) | Supports distal embolization as an independent/competing mechanism of downstream perfusion failure. |
| Mottahedin et al., 2023 (18) | Human and mouse ex-vivo metabolomics plus mouse tMCAO/reperfusion model with malonate intervention | Experimental/translational; not applicable | Controlled dose/intervention experiments | Primary experimental/translational analysis | Multiple cohorts; human ex-vivo time points n=4-8 and mouse n=4 in key metabolomics experiments; additional in-vivo cohorts | Supports succinate-associated metabolic reperfusion injury and a mechanism-adjacent metabolic intervention concept. |
| Bellomo et al., 2025 (27) | IMPreST cohort after successful EVT with BOLD-CVR | Prospective longitudinal cohort; analysis of included cohort | Nonrandomized | Secondary analysis of prospectively collected cohort data | 21 | Persistent steal physiology after angiographic success. |
| Tian et al., 2025 (35) | Single-center pre/post-MT CTP study | Retrospective | Nonrandomized | Primary retrospective analysis | 76 | Post-MT hypo-/hyperperfusion patterns and clinical associations. |
| Singer et al., 2026 (36) | STAMINA database DSA-PCC case-control analysis | Retrospective | Controlled observational | Secondary database analysis | 55 suspected microvascular failure + 55 stroke controls + 42 non-stroke controls | Angio-suite microvascular transit metrics after TICI 3. |
| Abraham et al., 2025 (37) | Multicenter MT follow-up imaging dataset analyzed with automated FIV/edema/HT/infarct-growth quantification | Retrospective imaging analysis of existing clinical data | Nonrandomized | Secondary imaging analysis | 986 available; 843 with complete data analyzed | Validates early follow-up imaging biomarkers as mechanistic/prognostic endpoints after MT. |
| ter Schiphorst et al., 2026 (13) | Single-center prospective LVO/EVT cohort with early post-reperfusion perfusion MRI and serial inflammatory biomarker sampling | Prospective observational cohort | Nonrandomized | Primary clinical imaging/biomarker analysis | 71 patients | Shows that early post-EVT perfusion deficits frequently reflect residual distal embolic/macrovascular abnormalities; visually defined microvascular hypoperfusion was uncommon. Provides a direct human framework for separating competing causes of post-reperfusion hypoperfusion. |
| Ng et al., 2026 (14) | Multicenter prospective observational study with serial post-thrombectomy perfusion MRI at approximately 2 h and 24-48 h | Prospective multicenter observational cohort | Nonrandomized | Primary clinical imaging analysis | 67 enrolled; 29 with eTICI 2c-3 analyzed for no-reflow | Demonstrates that imaging-defined no-reflow can persist, resolve, progress, or newly develop over time after technically successful reperfusion, supporting serial rather than single-time-point phenotyping. |
| CHOICE / Renu et al., 2022 (44) | 7-center phase 2b trial after successful thrombectomy | Prospective | Randomized, double-blind, placebo-controlled | Primary randomized-trial analysis | 121 randomized; 113 treated as randomized | Microcirculatory-rescue concept with intra-arterial alteplase; not glycocalyx-specific. |
| PEARL Investigators, 2025 (45) | 28-center trial after successful endovascular reperfusion | Prospective | Randomized, controlled | Primary randomized-trial analysis | 324 randomized | Supports clinical actionability of residual post-EVT dysfunction; mechanism not glycocalyx-specific. |
| Hou et al., 2025 (46) | Multicenter phase 1b/2a intra-arterial tenecteplase trial after successful reperfusion | Prospective | Phase 1b nonrandomized dose escalation; phase 2a randomized | Primary trial analysis | 205 total (48 phase 1b; 157 phase 2a) | Provides safety/dose-selection evidence for adjunct post-reperfusion lytic therapy. |
| Broekhuizen et al., 2010 (50) | Type 2 diabetes sulodexide clinical study | Prospective interventional study | Controlled; nonrandomized | Primary interventional analysis | 20 men (10 type 2 diabetes; 10 controls) | Cross-disease clinical evidence that systemic glycocalyx dimensions/permeability are modifiable; not acute stroke/MT. |
| Li et al., 2017 (51) | Rat carotid balloon-injury model | Experimental; not applicable | Randomized controlled animal experiment (4 groups) | Primary experimental analysis | 40 rats (4 groups, n=10 each) | Glycocalyx reconstruction with sulodexide in non-cerebral vascular injury. |
| Ying et al., 2023 (52) | Pediatric septic-shock cohort plus endothelial-cell and murine sepsis models | Prospective human cohort plus experimental studies | Human component nonrandomized; experimental models controlled | Primary human and experimental analyses | 28 children with septic shock; additional cell and mouse experiments | Cross-disease biomarker and glycocalyx-remodeling evidence; not cerebral/MT-specific. |
| Astapenko et al., 2025 (53) | Porcine enzymatic glycocalyx-damage pilot | Experimental pilot; not applicable | Controlled; 3 groups | Primary experimental analysis | 12 piglets (4 sulodexide-before injury; 4 sulodexide-after injury; 4 controls) | Non-cerebral large-animal glycocalyx modulation/microcirculation study; no specific post-MT efficacy inference. |
| Babić et al., 2026 (43) | Prospective AIS recanalization biomarker cohort comparing IVT, IVT+MT, and MT with serial peripheral sampling | Prospective observational | Nonrandomized; healthy control group | Primary clinical biomarker analysis | 60 AIS patients (three treatment groups) + 20 healthy controls | Serial syndecan-1, heparan sulfate, hyaluronan, and IL-18 measurements before and 24/48 h after therapy; reinforces treatment- and time-context dependence of circulating glycocalyx-related biomarkers. |
| Chen et al., 2022 (26) | Mouse photothrombotic MCAO with thrombolysis, MRI, fibrin-targeted imaging, histology, and capillary assessment | Experimental; acute/subacute | Randomized thrombolytic vs non-thrombolytic groups | Primary experimental analysis | 12 mice per ischemic treatment group; sham group n=6 | Demonstrates capillary fibrin deposition and stacked RBCs after recanalization/thrombolysis, providing primary experimental support for microvascular thrombotic no-reflow. |
| Costa et al., 2006 (20) | Rat transient MCAO/reperfusion model with systemic anti-C5 inhibition | Experimental ischemia-reperfusion | Controlled anti-C5 intervention; randomization not reported | Primary experimental analysis | Per-group sample sizes reported in source; no single total highlighted in abstract | Provides primary cerebral ischemia-reperfusion evidence that complement C5 contributes to inflammatory injury, lesion burden, and edema. |
| Shi et al., 2018 (28) | Anterior-circulation MT cohort with immediate post-treatment CT assessment of BBB disruption and 24-h hemorrhage imaging | Observational clinical cohort | Nonrandomized | Primary clinical imaging analysis | 210 patients | Direct human MT evidence that early BBB disruption is associated with hemorrhagic transformation and poor outcome, while remaining non-glycocalyx-specific. |
| Wang et al., 2026 (29) | Consecutive AIS EVT cohort integrating collateral status, CTP microcirculatory measures, BBB permeability, and HT outcomes | Retrospective clinical cohort | Nonrandomized | Primary clinical imaging analysis | 202 patients | Human EVT evidence linking microcirculatory dysfunction and BBB vulnerability with hemorrhagic transformation; supports an interacting vascular-injury framework rather than glycocalyx-specific causality. |

Abbreviations: AIS, acute ischemic stroke; ASL, arterial spin labeling; BBB, blood-brain barrier; BOLD-CVR, blood oxygenation level-dependent cerebrovascular reactivity; CBF, cerebral blood flow; CTA, computed tomography angiography; CTH, capillary transit-time heterogeneity; CTP, computed tomography perfusion; DSA, digital subtraction angiography; DSA-PCC, digital subtraction angiography parametric color coding; eTICI, expanded Treatment in Cerebral Ischemia; EVT, endovascular therapy; FIV, final infarct volume; HA, hyaluronan; HS, heparan sulfate; HT, hemorrhagic transformation; IMR, impaired microvascular reperfusion; IVT, intravenous thrombolysis; LVO, large-vessel occlusion; MRA, magnetic resonance angiography; MRP, magnetic resonance perfusion; mRS, modified Rankin Scale; MT, mechanical thrombectomy; NIHSS, National Institutes of Health Stroke Scale; sICH, symptomatic intracranial hemorrhage.
